# Diagnosis and Treatment of a Lateral Meniscal Cyst with Musculoskeletal Ultrasound

**DOI:** 10.1155/2015/432187

**Published:** 2015-02-05

**Authors:** Hamilton Chen

**Affiliations:** University of California, Riverside, Riverside, CA 92521, USA

## Abstract

Meniscal cysts are a relatively uncommon occurrence that may result in pain and disability in the knee. It is widely believed that meniscal cysts are secondary to fluid extrusion from a meniscus tear. Typically, diagnosis of a meniscal cyst typically requires magnetic resonance imaging (MRI) to delineate the cyst and any associated injuries. With improvements in sonographic technology, ultrasound has emerged as a sensitive modality for detection of meniscal cysts. We present a patient with a contraindication to MRI who was diagnosed with a lateral meniscal cyst by musculoskeletal ultrasound and treated with an ultrasound-guided lateral meniscal cyst aspiration and injection.

## 1. Introduction

Meniscal cysts are a relatively uncommon occurrence that may result in pain and disability in the knee. The incidence of meniscal cysts estimated in a prior magnetic resonance imaging study was 4% [1]. There are many possible etiologies for meniscal cysts, but it is widely believed that meniscal cysts are secondary to fluid extrusion from a meniscus tear [2]. Meniscal cysts will typically present with focal knee pain, swelling, and, on occasion, a palpable mass at the joint line.

Confirmation of the diagnosis of a meniscal cyst will usually require magnetic resonance imaging (MRI) due to its ability to delineate the cyst and evaluate any associated meniscal pathology. However, with the recent improvement in sonographic technology, ultrasound has emerged as a sensitive modality for detection of meniscal cysts [3, 4]. In addition, ultrasound may be used for therapeutic aspiration of the cyst [5]. We present a patient with a contraindication to MRI who was diagnosed with a meniscal cyst with musculoskeletal ultrasound and subsequently treated with an ultrasound-guided aspiration and injection of steroids.

## 2. Case

**Figure 1:**
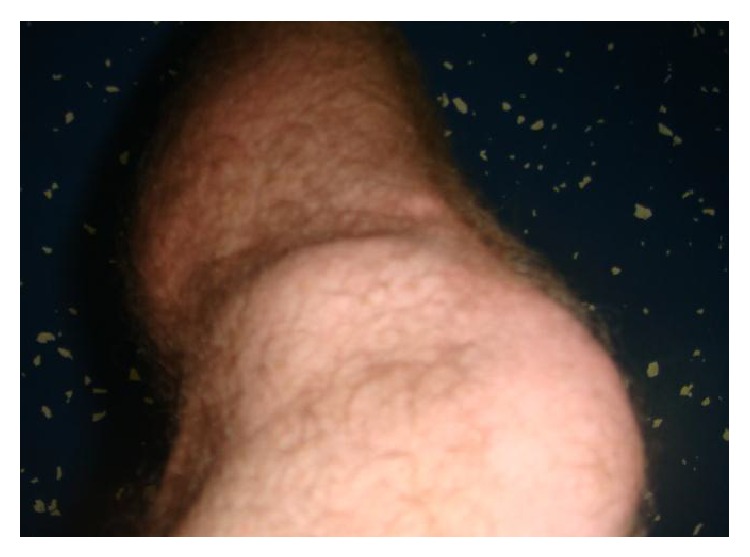
Visible mass on lateral joint line on the knee.

**Figure 2:**
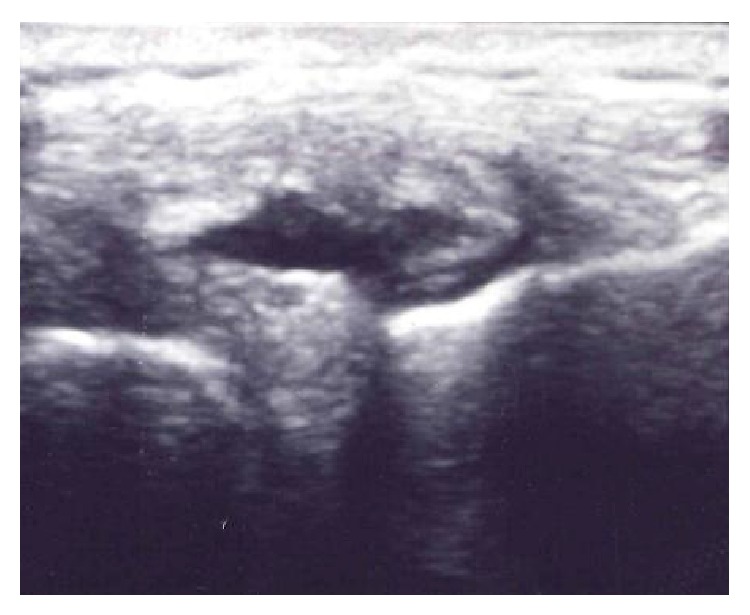
Ultrasound image in the coronal plane demonstrating an anechoic meniscal cyst (arrow) superficial to the lateral meniscus.

A 26-year-old male with no significant past medical history presented to our clinic with a chief complaint of left lateral knee pain. On examination, the patient had a visible 2 × 2 cm mass on the knee at the lateral joint line (Figure 1). The mass was tender to palpation. Mcmurray, Ober, Lachman's, and anterior/posterior drawer tests were negative. A diagnostic ultrasound was performed in clinic, which revealed an anechoic cyst extending from the lateral meniscus (Figure 2). The patient was provided with education regarding both conservative and surgical treatment options. The patient opted for nonoperative management of the meniscal cyst and proceeded with aspiration and injection of the cyst under ultrasound guidance.

**Figure 3:**
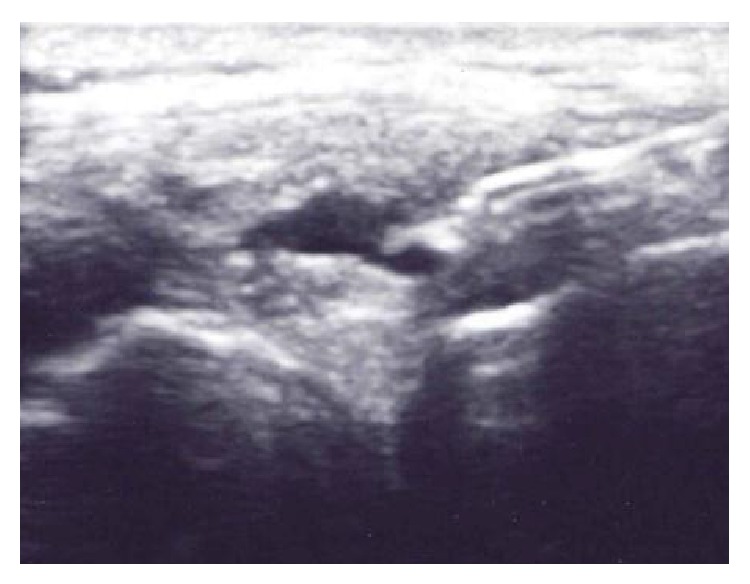
Ultrasound image in the coronal plane demonstrating aspiration of the meniscal cyst (arrow).

**Figure 4:**
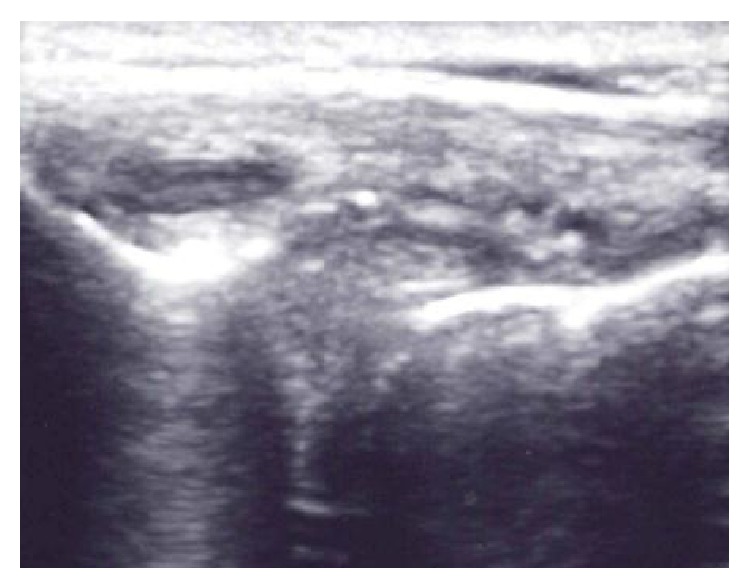
Ultrasound image in the coronal plane demonstrating resolution of the cyst immediately after aspiration and injection.

After obtaining informed consent, the lateral meniscal cyst was visualized using a Sonosite M-Turbo with the “hockey puck” 13–8 Mhz ultrasound probe. 1% lidocaine was administered subcutaneously and a 19 G needle was inserted into the cyst under direct ultrasound guidance in the long axis (Figure 3). The cyst was completely aspirated. Then, a solution consisting of 40 milligrams of methylprednisolone acetate (Depo-Medrol) and 1 mL of 1% lidocaine was injected. Follow-up scanning of the lateral joint line immediately after aspiration and injection revealed no visible cyst (Figure 4). The patient returned for follow-up 2 months after aspiration and injection of the cyst and did not experience recurrence of the meniscal cyst. The patient underwent a course of physical therapy and continued to do well at 6-month follow-up.

## 3. Discussion

Meniscal cysts are a rare diagnosis, with an estimated incidence of 1–8% in prior studies [6–8]. Typically, cysts are believed to have developed from extrusion of synovial fluid through a meniscus tear [7, 9]. Meniscal cysts may present as parameniscal cysts or intrameniscal cysts, based on the location of the cyst relative to the meniscus.

The diagnosis of a meniscal cyst is usually established through a combination of a good patient history, clinical examination, and diagnostic imaging. Patients with a meniscal cyst will typically report symptoms of focal knee pain and swelling along the joint line. Due to its association with meniscal cysts, symptoms of a meniscus tear, such as popping, joint stiffness, and locking may also be present. On examination, patients with meniscal cysts may often present with a palpable mass along the joint line. Parameniscal cysts of the lateral meniscus are more commonly palpable compared to parameniscal cysts of the medial meniscus [7]. Even though there are no “special tests” to specifically evaluate meniscal cysts, physical examination maneuvers to evaluate meniscal tears may be used due to the association of parameniscal cysts with meniscal tears.

MRI Imaging is typically considered the “gold standard” for a suspected meniscal cyst due to its ability to delineate the cyst and assess the menisci [10]. Moreover, there are often incidental findings of meniscal cysts on MRI in patients who are asymptomatic [6]. Despite the utility of MRI, it is expensive and patients may have contraindications for an MRI.

The availability of high-frequency and high-resolution ultrasound machines has made it possible for sonographic detection of meniscal cysts. Rutten et al. [11] demonstrated the sensitivity, specificity, and accuracy of ultrasound in the depiction of meniscal cysts as 97, 86, and 94%, respectively, with a positive predictive value of 94% and a negative predictive value of 92%. Sorrentino et al. found that high-resolution ultrasound had a sensitivity, specificity, PPV, and NPV of 94.23%, 100%, 100%, and 94.54%, respectively, for the detection of meniscal cysts [12].

High-resolution ultrasound has also allowed the possibility for image-guided aspiration of the meniscal cyst. Muddu et al. [13] first described the conservative treatment measure of meniscal cyst aspiration and injection following clinical palpation in 1992. Since then, there has only been 1 case series of 18 patients who underwent ultrasound-guided aspiration and injection of the parameniscal cyst [5]. Of these patients in the study, 10 patients had complete resolution of symptoms and 2 had a sustained satisfactory response to the procedure in the long term.

Our case is unique because it demonstrates the benefits of musculoskeletal ultrasound for diagnosis and treatment of a meniscal cyst when an MRI is contraindicated. Furthermore, the use of musculoskeletal ultrasound in the clinic on initial evaluation ensured efficient health care delivery, providing an accurate diagnosis and therapeutic plan on the same day without requiring a return trip for the patient to the clinic.

In conclusion, even though an MRI is the preferred modality for diagnosis of meniscal cysts, musculoskeletal ultrasound is a viable option for both diagnosis and treatment of this condition, especially in patients with contraindications to MRI. In addition, ultrasound-guided percutaneous aspiration and injection of painful meniscal cysts are a well-tolerated, simple, and effective procedure that may provide long-term symptomatic relief.
